# Large-Scale Event Extraction from Literature with Multi-Level Gene Normalization

**DOI:** 10.1371/journal.pone.0055814

**Published:** 2013-04-17

**Authors:** Sofie Van Landeghem, Jari Björne, Chih-Hsuan Wei, Kai Hakala, Sampo Pyysalo, Sophia Ananiadou, Hung-Yu Kao, Zhiyong Lu, Tapio Salakoski, Yves Van de Peer, Filip Ginter

**Affiliations:** 1 Department of Plant Systems Biology, VIB, Gent, Belgium; 2 Department of Plant Biotechnology and Bioinformatics, Ghent University, Gent, Belgium; 3 Turku Centre for Computer Science, Turku, Finland; 4 Department of Information Technology, University of Turku, Finland; 5 National Center for Biotechnology Information, Bethesda, Maryland, United States of America; 6 Department of Computer Science and Information Engineering, National Cheng Kung University, Tainan, Taiwan; 7 National Centre for Text Mining, School of Computer Science, University of Manchester, Manchester, United Kingdom; University of Leuven, Belgium

## Abstract

Text mining for the life sciences aims to aid database curation, knowledge summarization and information retrieval through the automated processing of biomedical texts. To provide comprehensive coverage and enable full integration with existing biomolecular database records, it is crucial that text mining tools scale up to millions of articles and that their analyses can be unambiguously linked to information recorded in resources such as UniProt, KEGG, BioGRID and NCBI databases. In this study, we investigate how fully automated text mining of complex biomolecular events can be augmented with a normalization strategy that identifies biological concepts in text, mapping them to identifiers at varying levels of granularity, ranging from canonicalized symbols to unique gene and proteins and broad gene families. To this end, we have combined two state-of-the-art text mining components, previously evaluated on two community-wide challenges, and have extended and improved upon these methods by exploiting their complementary nature. Using these systems, we perform normalization and event extraction to create a large-scale resource that is publicly available, unique in semantic scope, and covers all 21.9 million PubMed abstracts and 460 thousand PubMed Central open access full-text articles. This dataset contains 40 million biomolecular events involving 76 million gene/protein mentions, linked to 122 thousand distinct genes from 5032 species across the full taxonomic tree. Detailed evaluations and analyses reveal promising results for application of this data in database and pathway curation efforts. The main software components used in this study are released under an open-source license. Further, the resulting dataset is freely accessible through a novel API, providing programmatic and customized access (http://www.evexdb.org/api/v001/). Finally, to allow for large-scale bioinformatic analyses, the entire resource is available for bulk download from http://evexdb.org/download/, under the Creative Commons – Attribution – Share Alike (CC BY-SA) license.

## Introduction

The richness of information available in the vast biomedical literature has motivated many studies and resources to include textual data as an information source [1]–[4]. However, applied text mining algorithms have often relied on relatively simple text analysis or have been designed to cover only a specific domain, organism, or relation type.

**Figure 1:**
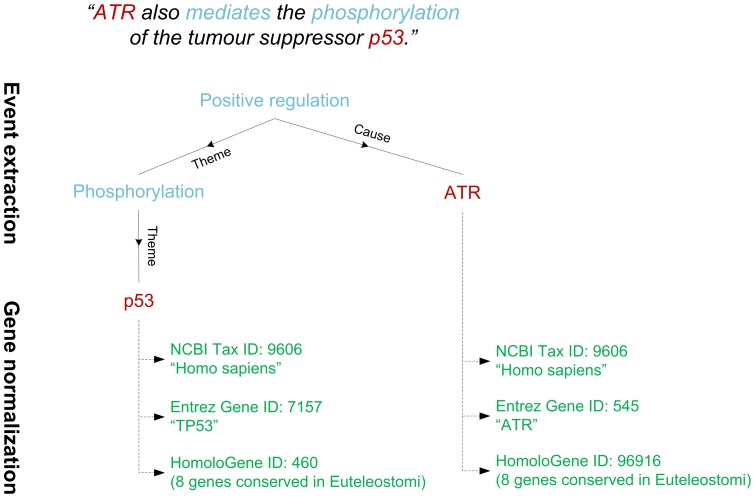
Illustration of event extraction and gene normalization. The gene mentions recognised in text are in red and the extracted event structures in blue. The normalization algorithm further maps the ambiguous gene mentions to unique database identifiers (in green).

During the last decade, the development of fully automated text mining techniques has attracted wide interest, resulting in several general-purpose stand-alone text mining tools [5]–[11]. In this context, text mining involves two key challenges: the automated extraction of formal representations of statements from text, and the identification of the real-world objects, such as genes and proteins, that these statements refer to (Figure 1). In fundamental research on biomedical natural language processing, these challenges have been addressed largely independently, with one major line of research focusing on event extraction and another on gene name normalization.

*Event extraction* refers to the automated extraction of structured representations of biological processes, or “events”, from text. Extracted event structures are typed associations of arbitrary numbers of participants – analogous to reactions in pathway representations such as SBML and BioPAX [12], [13] – and cover fundamental molecular processes such as binding and phosphorylation, their regulatory control, and the identification of specifically negated statements as well as contextual information such as cellular locations [14]. Biomedical event extraction was popularized by the BioNLP Shared Task (ST) on Event Extraction of 2009 [15]. Recently, the scope of event extraction has been further broadened for epigenetics, post-translational modifications, and core protein information such as protein domains and complexes [16], [17].

*Gene normalization* is the task of uniquely identifying the biological entities that gene symbols in text refer to, normally cast as associating text strings to database identifiers. Due to the high degree of both inter-species and intra-species gene name ambiguity [18], this task is critically important for assuring the applicability of text mining information in real-world applications. To connect ambiguous symbols, abbreviations and synonyms in text to unique identifiers such as Entrez Gene IDs [19], gene normalization algorithms resolve ambiguities by using contextual information found in the document. As a result, text mining information can be directly linked to the vast bioinformatics resources available on known genes and proteins, including databases at NCBI Entrez [19], UniProt [20], KEGG [21] and PDB [22]. Gene normalization has been a major focus of BioCreative, the longest-running community-wide challenge in the domain [23]–[26].

In this work, we join together the two independent lines of research on event extraction and gene normalization by combining two state of the art systems from the BioNLP Shared Task and the BioCreative challenge. Integrating these approaches with a previously released gene family assignment algorithm, we further broaden the normalization scope to cover not only gene and protein identifiers, but also more general gene families that group evolutionarily related and functionally similar genes across species. Additionally, we present and evaluate a novel normalization algorithm using canonical symbols and taxonomic assignment. Our analyses illustrate that these different normalization algorithms exhibit different properties, and we demonstrate how they can be used to complement each other, providing a powerful means to query information in the literature at varying levels of detail. All methods are run on all PubMed (PM) abstracts and PubMed Central (PMC) open access full texts, resulting in a unique dataset for text mining researchers, bioinformaticians and biologists. A novel API allows customized querying of this data in a variety of applications.

In comparison to previous large-scale analyses [10], [11], this study presents a significant extension in the semantic scope of the event extraction data, and additionally supports a novel multi-layer approach to gene normalization through the combination of canonical forms, gene families and gene IDs.

## Materials and Methods

**Figure 2:**
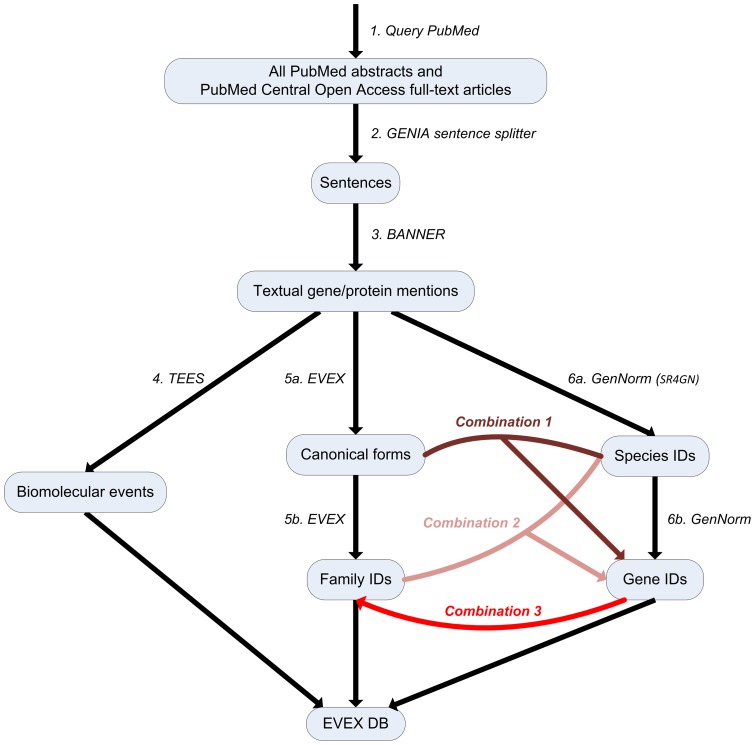
Overview of the various steps and programs involved in this study. The black arrows represent previously published tools, which have all been integrated in this study to create a unified text mining pipeline. Furthermore, the various opportunities for combining the different methods for gene normalization are presented by the colored edges and discussed in detail in the text.

The text mining pipeline applied to all PM abstracts and PMC full-text articles consists of several consecutive steps. After downloading and pre-processing all data from the source literature databases and identifying the sentences in all articles, the first step towards information extraction entails the recognition of gene and protein mentions in text. Next, event extraction is performed to detect statements of biological processes and regulatory associations that involve the mentioned genes and proteins. Then, a gene normalization step is applied to resolve the ambiguous gene symbols to database identifiers. Finally, all data is integrated to extend our previously introduced resource, EVEX, for custom browsing and querying. A general overview of the text mining pipeline is depicted in Figure 2 and explained in detail in the next sections.

### Text pre-processing

While PM abstracts can be processed relatively straightforwardly, we have implemented a few novel pre-processing steps to extract information from full-text PMC articles. First, we apply a Unicode-to-ASCII mapping, building on tools introduced for the ST'11 [27], and the NLM Lexical Variant Generator toAscii tool. This conversion is reversible, allowing analyses created by the system to be mapped back into the source XML. Further, we annotate all information extracted from full-text articles with the specific section the data was retrieved from, such as “Abstract”, “Introduction” “Results” and “Methods”, allowing for custom filtering of the data by article section.

### Entity recognition

We perform the detection of gene and protein mentions in text (Figure 2, step 3) with the BANNER named entity recognition system [28]. Recent releases of BANNER are competitive with the state of the art at the standard BioCreative 2 gene/protein mention recognition task, achieving an F-score of 86.4%. Following the setting of this task, BANNER identifies specific spans of text referring to genes, proteins, and related entities such as protein complexes, but does not differentiate between these entity types nor involve any form of normalization.

### Event extraction

For event extraction (Figure 2, step 4), we apply the Turku Event Extraction System (TEES), originally developed for the BioNLP ST of 2009 [15]. Since its initial release, we have considerably extended TEES to address the BioNLP ST'11 [29] and added tool wrappers for the GENIA Sentence Splitter [30], the McClosky-Charniak-Johnson parser for syntactic analysis [31], [32], the Stanford tools [33], and the BANNER named entity recognizer [28], creating a fully integrated, standalone application. TEES has also undergone major revisions to ensure easier usage of the system by third parties. Finally, TEES was retrained on the BioNLP ST'11 GE corpus, 30% of which is sourced from PMC full-text documents [16].

We previously applied the original version of TEES to 19 million PM abstracts to produce the first large-scale event dataset [10], [34], covering all event types of the GENIA event extraction (GE) task of the ST'09 (Table 1). In the current study, we bring this dataset up-to-date with the latest results of the PM abstracts of 2011 and 2012 and additionally process the whole body of available full-text articles from the PMC open access subset, thus effectively doubling the size of the analysed text. Additionally, we fully integrate data from recent large-scale extraction runs covering two novel event extraction challenges of the ST'11: Epigenetics and Post-translational Modifications (EPI) and entity relations (REL) [35] (Table 1). The addition of event types from the Shared Task 2011 results in an event extraction resource of unprecedented semantic scope. Further, as TEES achieved high performance at the ST'11, with first rank at both the EPI and REL tasks, the quality of these analyses is assured to represent the state of the art.

**Table 1:** Event types processed in this study.

	Event types	Example text fragment	Count
	Transcription	during meiosis, **transcription** of the EXO1 gene is highly induced	561K
	Gene expression	DUN1 was not identified as differentially **expressed** in tlc1Delta strains	10453K
ST'09	Localization	the subcellular **localization** of endogenous ICAD was examined	1805K
	Protein catabolism	hyperglycemia leads to CREB protein **degradation** in vivo	279K
ST'11	Binding	in vitro **binding** of GrpLSH2 domain to tyrosine-phosphorylated SHP-2	6154K
(GE)	Regulation	the **effect** of the opiate (heroin) on DARPP-32 expression	3194K
	Positive regulation	p27 (Kip1) **enhances** myelin basic protein gene promoter activity	9955K
	Negative regulation	miR-198 functions to **repress** Cyclin T1 protein expression	6010K
	(De)phosphorylation	mutants were **phosphorylated** significantly less than the wild-type	1005K
	(De)hydroxylation	reduced Hif1alpha **hydroxylation** at both Pro402 and Pro564 residues	16K
	(De)ubiquitination	**polyubiquitination** of Shank and GKAP increased after retrieval	89K
ST'11	DNA (de)methylation	NGFI-A binding to its consensus sequence is inhibited by **DNA methylation**	173K
(EPI)	(De)glycosylation	TibA was the first **glycosylated** AT described in *E. coli*	172K
	(De)acetylation	in human myocytes where over-expressed Sir2 **deacetylates** H2A.Z	135K
	(De)methylation	increased **trimethylation** of histone H3 (H3K27me3) on the IL-12B promoter	147K
	Catalysis	an enzyme that **catalyzes** the ATP-dependent phosphorylation of IP	43K
ST'11	Protein-Component	truncation of the **C-terminal domain** of FrdD	2178K
(REL)	Subunit-Complex	the *E. coli* cheB and cheY **gene complex**	681K

All event types included in this study, their counts, and example text fragments. Phosphorylation is only listed once, but was originally also included in the ST'09 and ST'11 GE data. The EPI types, with the exception of Catalysis, specifically include a positive and a reverse variant.

To rank the event predictions according to their reliability, TEES assigns a confidence score for each classification step. These scores are aggregated to normalized event scores using the *minimum* function, thus requiring all components of an event to be confidently predicted for the event to be given a high score [36]. Within this study, these scores are renormalized to a [0,6] scale, with the average event confidence equal to 3 and avoiding counter-intuitive negative values for confidence scores. In contradiction to previous work, we have applied this renormalization to every combination of event type, number of GGP arguments, and number of event arguments separately, to account for the variance in predicted scores for different general event structures.

### Gene normalization

We previously released a large-scale event extraction dataset covering only abstracts and the event types of the ST'09 as part of the EVEX database [10]. At the time, we did not have access to a normalization algorithm that could link the ambiguous gene mentions to unique gene identifiers. Instead, canonical forms of the mentions were produced (Figure 2, step 5a), and a novel gene family assignment algorithm was implemented (Figure 2, step 5b). In the current study, we augment these approaches by a recently released state of the art normalization system (Figure 2, step 6a–6b), and we further exploit the complementary nature of these different methods to extend and improve on both the family assignment as well as the ID normalization algorithm (Figure 2, Combination 1–3). An overview of the different techniques is presented in Table 2. They are described in more detail in the next three sections and strategies for their combination in the section following these.

**Table 2:** Normalization methods and their properties.

	Lexical variation	Synonymy	Orthology	Species-specific
Canonicalization	yes	no	no	no
Family assignment	yes	yes	yes	no
Gene normalization	yes	yes	no	yes

The different normalization methods applied in this study, and whether or not they account for lexical variation, synonymy, orthology and species-specific resolution. By creating combinations of these algorithms, their individual strengths can be aggregated.

#### Canonical forms

The canonicalization algorithm as previously implemented within the EVEX resource has two main goals. First, it resolves small lexical variations in spelling, mapping both “*Esr-1*” and “*ESR 1*” to the same canonical form “*esr1*”. Secondly, it resolves and removes commonly used prefixes and suffixes, mapping also the noun phrase “*human Esr-1 gene*” to “*esr1*”. For details on the original suffix striping algorithm we refer to [10]. These canonical forms provide a powerful way to query textual representations of events through symbol search, transparently dealing with lexical variation of gene symbols. However, as the canonicalization algorithm does not resolve synonymy or the species of gene mentions, it does not alone allow for a reliable mapping to database identifiers.

#### Family assignment

The original EVEX release additionally included a family assignment algorithm, resolving the canonical symbols to the most plausible gene family as defined by HomoloGene (eukaryotes, [19]), Ensembl (vertebrates, [37]) or Ensembl Genomes (metazoa, plants, protists, fungi, and bacteria, [38]). The reasoning behind the family-based assignment is that homologous genes, evolved from a common ancestor, often still exhibit similar functional behavior, and are consequently assigned to similar names in genome annotation projects. For example, the human *Esr-1* gene is known to be involved in breast cancer, while the mouse *Esr-1* gene has been connected to tumorigenesis. In some cross-species studies, it may even be impossible to deduce, for a given textual mention, which exact gene is meant by the authors, and some statements may involve general descriptions applying to all genes contained within one family.

The original family assignment relies solely on the canonical forms of the gene symbols to determine the most plausible gene family to a specific gene mention. Disambiguation between different candidate gene families is performed by selecting the family that contains the most genes with this specific canonical form as synonym. In practice, this results in the interpretation of “*esr*” as the family of *estrogen receptor* genes, rather than the less common usage of this abbreviation for e.g. *Enhancer of shoot regeneration*. Consequently, this method is prone to errors when a gene symbol is shared by functionally different genes [10]. However, by mapping several different canonical forms to the same gene family, this algorithm does successfully deal with common synonymy.

#### Gene normalization

To provide even more detailed normalization, in this study we apply the GenNorm normalization algorithm [39], [40] for assigning organism-specific Entrez Gene identifiers to the textual gene mentions. GenNorm is an integrative method for cross-species gene normalization and achieved the first rank by several evaluation criteria in the gene normalization task of the BioCreative III Challenge [41]. To ensure straightforward integration with the event extraction pipeline and other data in EVEX, we have adjusted GenNorm to work with the entity mentions detected by BANNER for event extraction, instead of running its own entity recognition module.

As GenNorm has been developed in the context of the BioCreative III task, it produces document-level normalization, i.e. a set of Entrez Gene identifiers relevant to each input document. This is achieved by first identifying one or many candidate normalizations for each gene mention in the text. Subsequently, these candidate normalizations are aggregated on the document level to produce a final set of normalizations, consistent across the article. However, in order to integrate GenNorm results with the event analyses, mention-level gene normalizations are needed. We thus extended GenNorm to revisit the original per-mention candidates and to choose for each one the candidate most consistent with the document-level set.

For gene mentions where full resolution into an Entrez Gene identifier is not possible, GenNorm still assigns the most likely organism of the mention, using its stand-alone open source module SR4GN [42]. SR4GN was proven to achieve state of the art results, reporting 85.42% in accuracy.

#### Combining normalization strategies

The three normalization algorithms described above exhibit different properties (Table 2) and their complementary nature can be exploited in a combined approach. For example, the GenNorm results can be used as the priority choice for resolving the genes and proteins extracted from text to gene families (Figure 2, Combination 3). To this end, we query the EVEX database to determine whether the gene ID, assigned by GenNorm, is linked to a known gene family, and assign that family accordingly. For cases where the GenNorm algorithm fails to produce a unique gene identifier, we have implemented an adapted version of the original symbol-based family assignment procedure as a fallback mechanism. Taking into account both the canonical form of the gene mention, as well as the organism assigned by GenNorm, the new algorithm tries to assign a family that contains at least one gene of the specified organism. When there are multiple candidate families, the family is picked that contains the most genes with this specific canonical form as synonym. If there is no candidate family matching the organism assignment, the original symbol-based algorithm is applied.

This novel approach can improve the accuracy of the family assignment by using the newly introduced mention-level gene normalizations and taxonomic assignments, taking into account the context of the full document. Conversely, the family assignments can assist in extending the coverage of the gene normalization algorithm itself (Figure 2, Combination 2). In cases where the inter-species ambiguity of a specific gene symbol is too high, GenNorm will not assign a unique gene identifier, but the fallback procedure of the family assignment may still assign a family to the mention. When this family contains a gene of the organism determined by GenNorm, we can transfer the ID of this gene to the mention, effectively resolving the inter-species ambiguity through the gene families. One final method of assigning unique gene IDs, is through the combination of canonical symbols with taxonomic assignments (Figure 2, Combination 1). While inter-species ambiguity can not be resolved through this simple approach, a significant proportion of gene symbols are unique within one species and can thus be assigned based solely on the canonical symbol and species.

The effects of combining these different methods for both family as well as gene ID assignment are detailed in the Results and Discussion Section.

### Data retrieval

All the data we have generated in this study are made publicly available through bulk downloads, an upgraded version of the EVEX web application, and through a novel programmatic interface, which allows custom querying of both the event structures and the normalization data. We have invested substantial engineering efforts into assuring that this large dataset can be efficiently queried, providing real-time response times even for queries involving complex structures occurring tens of thousands of times in the data. We solicit community feedback on both the website and the API, as these resources will be closely maintained and further improved upon in future efforts.

## Results and Discussion

### Extraction statistics

We have run all methods detailed in the previous section on all 21.9 million PM abstracts and 460 thousand PMC open access full-text articles (data downloaded on June 25, 2012). To make the processing times manageable in practice, the pipeline was parallelized over more than a hundred cluster machines, enabling the processing of all data in a matter of days. Analysing the total run time, the most time-consuming tasks in the pipeline are the syntactic analysis (41%), gene recognition (8%), gene normalization (35%) and event extraction (14%).

**Figure 3:**
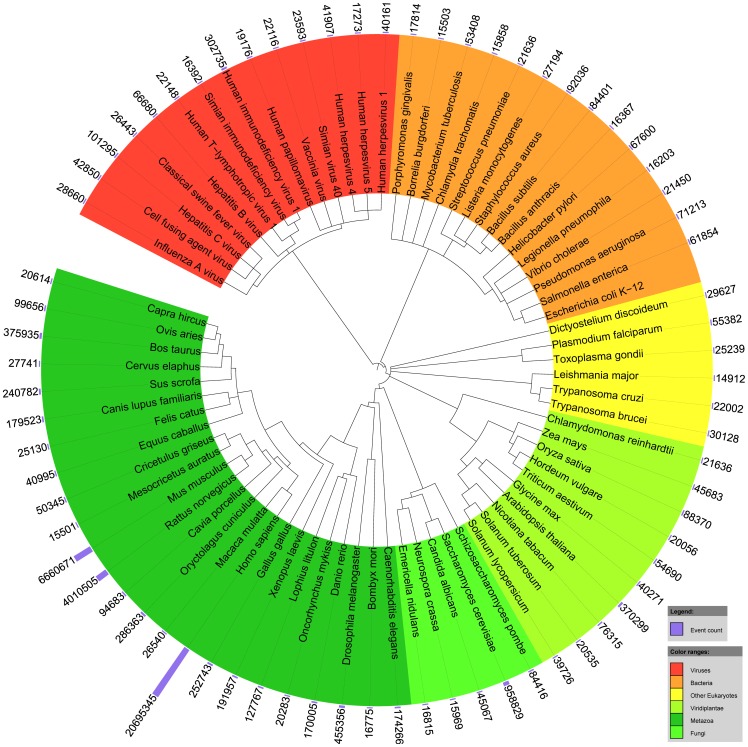
The most frequently occurring organisms, by the number of associated events found in literature. This plot illustrates that this study covers normalized event data across all domains and kingdoms. It was created with iTOL [51], and the phylogenetic tree is constructed through the information available at NCBI Taxonomy [19].

The automated processing of all available PM abstracts and PMC full-texts yielded more than 40 million detailed biomolecular events among 76 million gene/protein mentions (Table 3). These mentions were subsequently normalized to more than 120 thousand distinct genes from over 5000 species across the full taxonomic tree, including viruses, bacteria, fungi, plants and animals (Figure 3). Resolving these genes to families, ca. 28,700 HomoloGene families, 28,900 Ensembl families and 50,700 distinct Ensembl Genomes families were found.

**Table 3:** Overview of extraction statistics.

	Abstracts	Full text	Total	Entrez Gene	Ensembl Genomes
Articles	6.4M	384K	6.8M	–	–
Sentences	54.8M	66.9M	121.6M	–	–
Gene/protein mentions	43.3M	33.3M	76.5M	28.8M (37.6%)	47.9M (62.6%)
Events	23.5M	16.7M	40.2M	16.3M (40.5%)	26.0M (64.7%)

Extraction statistics for PubMed abstracts and PubMed Central full texts with at least one identified gene/protein mention. The last two columns state the number of mentions/events that could be fully normalized to Entrez Gene identifiers or Ensembl Genomes families.

The various normalization strategies can be combined with the event extraction results by defining equality of different event occurrences across documents. For instance, two events can be regarded as equal when they have the same event structure and their arguments pertain to the exact same gene identifiers. Using this definition, the number of instances in the data can be reduced by grouping similar statements together. However, not all events can be fully normalized using Entrez Gene IDs, as some of the participating arguments may not have been assigned to gene identifiers. Out of the original set of 40.2 million events, we were able to map 16.3 million (40.5%) to unique identifiers, together resulting in a smaller set of 1.5 million unique normalized events (Table 3).

The equality of events may alternatively be defined through the gene families, regarding two events as equal when they have the same structure and involve entities from the same gene families. This definition groups together interologs, conserved interactions between homologous pairs of proteins, and can thus support comparative genomics use-cases. Out of the original set of 40.2 million events, we linked 26.0 million events (64.7%) to gene families from Ensembl Genomes, a significantly higher fraction than that linked to Entrez Gene (Table 3). This result also holds for HomoloGene and Ensembl families, and demonstrates that a combined normalization procedure can significantly improve recall by using the family assignment of a gene/protein symbol when no Entrez Gene ID could be determined. More detailed analysis of normalization combinations are provided in the section on Normalization performance below.

### Abstracts vs. full texts

The addition of full-text event extraction significantly increases the coverage of information in biomolecular text, nearly doubling the text mining dataset in size compared to using PubMed abstracts only (Table 3). We have found that on average, full texts contain only 25 events per 100 sentences, compared to 43 events in 100 sentences from abstracts. These numbers confirm that full texts are more sparsely populated with information on biomolecular processes than abstracts, supporting the findings of earlier, smaller scale evaluations [43].

To further assess the added value of processing full-text articles rather than just abstracts, we analysed the retrieval of equivalent events within and across articles, defining events as equal when their event structure is equivalent and their arguments pertain to the same Entrez Gene identifiers. We found that only 7% of all events extracted from the body of a full-text PMC article could also be found in its abstract, results that are in line with previous reports [44]. When not limiting the search to the abstract of the same document, but including all available PM and PMC abstracts, we found that still only 37% of the events from full texts could be retrieved from any abstract. It is thus clear that the full texts contain a wealth of information that can not be extracted by only processing abstracts.

### Event extraction performance

The event extraction algorithm of TEES was previously evaluated in the framework of the BioNLP Shared Tasks [15], [45], measuring precision, recall and their harmonic mean, the F-score. In the latest Shared Task of 2011, TEES achieved top-ranking performance for the GE, EPI and REL tasks, achieving F-scores between 53% and 58% [29].

To establish whether these official benchmark results, evaluated on small domain-specific corpora, can be extrapolated for event predictions over the entire literature, we have manually evaluated a sample of predicted events to determine the precision rate of the extraction system. To this end, a random set of 100 events was taken from PMC article bodies and PM/PMC abstracts each. Recall was not evaluated as this requires full annotation of the evaluation documents, an extremely time-consuming task.

**Figure 4:**
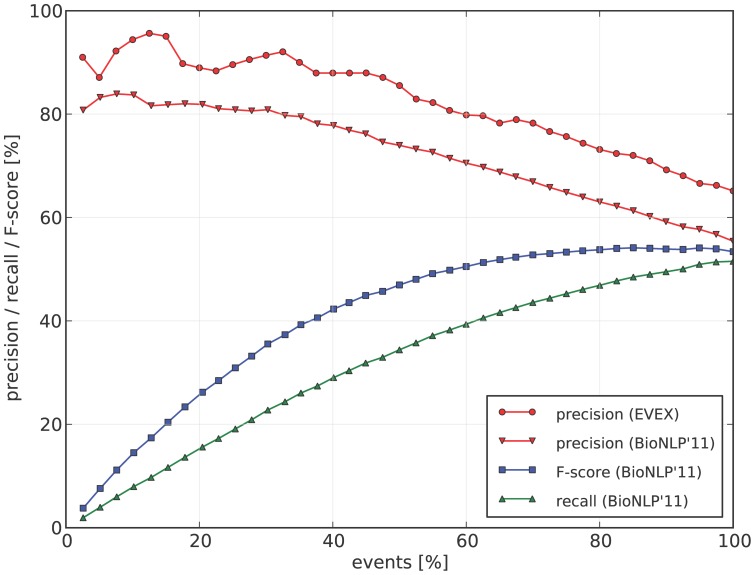
Event extraction performance. Both the evaluations of the BioNLP ST'11 GE task development set (3021 events, ST evaluation scripts) as well as a fully random sample (200 events, manually evaluated) are depicted. Events are ordered by their confidence scores, and plotted at different precision/recall trade-off points.

Despite the relatively small scale of this evaluation, the observed general trend is well in line with the official results (Figure 4, precision curves), which indicates that the method generalizes well. The slightly higher precision numbers of the manual evaluation, compared to the gold standard results, support a finding reported in the literature previously [17]. Finally, the precision of events extracted from abstracts (68%) was found to be higher than those in article bodies (62%), confirming that mining full-text is more difficult, as previously determined by the ST'11 GE results [16].

By ranking events using the automatically assigned scores, subsets of the event data can be selected with higher precision at the cost of lower recall. We observe the relation between precision and confidence scores both on the ST data and the manual evaluation (Figure 4). As measured on the ST data, e.g. 80% precision corresponds to 25% of all events, which still translates to 8.6M high-precision events.

The full set of events extracted within this study has an average confidence score of 3. When looking at the subsets of events that can be normalized into either Ensembl Genomes families or Entrez Gene identifiers, the average confidence value of the events rise to 3.06 and 3.09 respectively. Both these differences are statistically significant (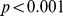).

Finally, note that the event extraction step is limited by its aim to only extract events within single sentences, not crossing sentence boundaries. It was previously determined that the amount of intersentence events is between 6–9% of all data [29]. In future work, this limitation could be resolved by accurately including coreference resolution data [46], a task which will be fully integrated within the event extraction challenge of the BioNLP Shared Task 2013 (http://bionlp.dbcls.jp/redmine/projects/bionlp-st-ge-2013).

### Performance of assigning gene identifiers

The GenNorm algorithm was thoroughly evaluated through the BioCreative III challenge, which uses Threshold Average Precision (TAP) scores. The gold standard BioCreative test set consists of 50 PMC full-text articles manually annotated with Entrez Gene identifiers [26]. Among the BioCreative III participants, GenNorm achieved the highest TAP scores when evaluated on this gold standard, obtaining values between 0.32 and 0.36 [41]. Translated to standard metrics, this corresponds to a precision of 56%, a recall of 40% and an F-measure of 47% [40].

To assess the performance of the GenNorm algorithm in combination with the canonicalization and family assignments (Figure 2, Combination 1–2), we perform additional evaluations on the BioCreative gold standard normalization dataset. To this end, we extracted from the results of our large-scale run the subset of articles that are included in the BioCreative dataset. For GenNorm, the results were notably lower than previously reported: 38.1% precision, 26.9% recall, and 31.5% F-measure. We unveiled the underlying causes through manual inspection of this discrepancy. First, the normalization effort in this study is based on the BANNER entity recognition software, trained on the GENETAG corpus [47], which includes not only the recognition of genes and proteins, but also of a broader range of biological entities such as families and complexes. While this property of BANNER is desirable for our goal of also extracting gene families from text, its broad scope may cause errors for the GenNorm normalization system, which was developed to only resolve gene and protein mentions.

However, by far the most important cause for the drop in performance is the fact that the event extraction pipeline does not process figures or tables. Indeed, such input data can not be processed by standard event extraction techniques, and has thus been *a priori* removed from further analysis. Consequently, the document-level set of Entrez Gene identifiers, as defined by the BioCreative dataset, contains many identifiers that fall out of scope of our text mining pipeline. For example, more than 100 gene identifiers from the full-text article PMC2887815 originate from a table, resulting in a large number of false negatives in our evaluation. Furthermore, the identifiers mentioned in tables are not available for ensuring document-level consistency by GenNorm, resulting in even more errors. For these reasons, we use the F-measure of 31.5% obtained with GenNorm applied within the context of the event extraction pipeline, as the baseline for our other methods in this study. We regard the extension of our text mining algorithms to additionally include tables and figures as interesting future work.

Table 4 details the performance of the different normalization strategies on the BioCreative test set articles. Surprisingly, the updated canonicalization algorithm using the taxonomic assignments of GenNorm (Figure 2, Combination 1) works almost as well as GenNorm itself, and even outperforms GenNorm on precision. This result suggests that intra-species ambiguity on the BioCreative dataset is relatively low. An integrative approach taking this method as a priority choice and GenNorm-assigned IDs as a fallback mechanism (Canonical + GenNorm) improves on this further, even outperforming GenNorm with 32.2% F-score.

**Table 4:** Performance of the various normalization algorithms.

	Precision	Recall	F-score
GenNorm	38.1	26.9	31.5
Canonical	**41.8**	**24.9**	**31.2**
HomoloGene	27.6	19.2	22.7
Ensembl	29.5	10.7	15.7
Ensembl Genomes	31.1	20.7	24.9
Canonical + GenNorm	35.3	29.8	**32.3**
GenNorm + Ens. Genomes	33.0	30.4	31.7
Canonical + GenNorm + Ens. Genomes	32.4	**31.4**	**31.9**

Performance of the various algorithms for Entrez Gene identifier assignment, as measured on the BioCreative III dataset. The canonical and family assignment algorithms both refer to the combined procedure which use the taxonomic assignments by GenNorm to enable species-specific ID disambiguation (Figure 2, Combination 1–2).

Evaluating the ability of the gene family assignments for determining unique, species-specific gene identifiers (Figure 2, Combination 2), we notice that Ensembl Genomes outperforms the other two family definitions. This finding is most likely attributed to the more general scope of Ensembl Genomes, covering a wider range of organisms across the taxonomic tree. However, by itself this method still only achieves 24.9% in F-score. Further integrating this method as a fallback mechanism for the GenNorm algorithm (GenNorm + Ens. Genomes), it achieves 31.7% in F-score, obtaining comparable results to the original GenNorm performance, but with a better balance between precision and recall. When constructing a 3-layered fallback mechanism, taking the canonicalization algorithm as priority choice on top of this combination (Canonical + GenNorm + Ens. Genomes), an F-score of 31.9% is achieved. While this is not an improvement over the two-layered Canonical + GenNorm approach, we do notice that this method achieves the highest recall.

These analyses illustrate the added value of using the canonicalization algorithm on top of the GenNorm predictions, producing the highest possible performance within these task settings. While the gene families can not be used to further improve on this combination in general, their biggest contribution lies in the fact that they are able to increase recall and cover more event occurrences. In future work, we plan on further evaluating these opportunities on the CRAFT corpus, a recently released mention-level gene normalization dataset [48].

### Performance of assigning gene families

A multi-level approach to normalization is applicable not only to the identification of unique genes for textual mentions, but also to the assignment of families. Considering the fact that GenNorm achieves higher precision compared to the EVEX family assignment algorithm (Table 4), we can use the GenNorm results as a priority choice for family assignment (Figure 2, Combination 3), with the EVEX algorithm serving as a fallback procedure. To evaluate the influence of this new approach, we again turn to the BioCreative III test set. We have mapped the gold-standard Entrez Gene identifiers in this dataset into their correct gene families using the three gene family definitions applied in EVEX: HomoloGene, Ensembl and Ensembl Genomes. While 82% of all gold-standard genes could be mapped into a family in at least one of the family definitions, only 29% are covered by all three, illustrating the complementary nature of the family definition resources. The mapping to Ensembl Genomes results in the largest gold standard dataset of document-level family assignments, with 921 true positives. The datasets for HomoloGene and Ensembl contain respectively 545 and 632 true positives.

Table 5 presents the performance of the various algorithms for gene family assignment for Ensembl Genomes. The original EVEX method (row 1) is compared to the adapted version implemented in this study (row 2), using the taxonomic identifiers to improve disambiguation between candidate families. We notice a slight improvement for this adapted family assignment algorithm, with F-score increasing from 26.0% to 27.5%. Further creating a novel combined approach (row 3), assigning gene families using the GenNorm system when it produces a normalization, and by the adapted EVEX method otherwise, this method outperforms the EVEX family assignment on all measures (F-score of 29.7%), demonstrating that the integrative normalization approach notably improves the quality of gene family assignment. Similar results are obtained for the other two family definitions, though the reported F-scores in these evaluations are significantly higher, with a combined F-score of 34.3% and 35.7% for HomoloGene and Ensembl, respectively. However, these family assignments exclude non-eukaryotic species, resulting in an easier task to resolve. A possible alternative strategy would be to use only GenNorm for family assignment, without providing a fallback mechanism when no normalization is present (Table 5, last row). This would have resulted in a higher total F-score of 38.8%, but at a considerable loss of recall, which would translate to the loss of family mapping for 8.5 million events.

**Table 5:** Performance of the gene family assignment algorithm.

	Precision	Recall	F-score
EVEX (original)	18.9	41.9	26.0
EVEX (adapted)	19.8	46.5	27.5
EVEX (adapted) + GenNorm	21.5	**47.7**	**29.7**
GenNorm	**41.5**	**36.5**	**38.8**

Performance of the gene family assignment using Ensembl Genomes definitions, as measured on a modified version of the BioCreative III dataset, translating gold gene IDs to their correct families. The last row depicts family assignments based on GenNorm only.

When interpreting these results, it is important to realise that this evaluation is performed on a dataset of family assignments, created as such by translating the manually annotated gene identifiers to families. In fact, mentions such as “the Esr-1 family” are out of scope for the BioCreative III normalization task and thus not annotated in this corpus. When the family assignment correctly determines the correct family ID for such a mention, this will show up as a false positive in our evaluation, artificially lowering precision rates. However, as there is, to the best of our knowledge, no other suitable mention-level gold standard dataset for evaluating gene family assignment in text, we accept these BioCreative III results as broadly indicative of recall and lower-bound estimates of precision.

To further investigate the added value of using the original family assignment as a fallback mechanism, we have evaluated this algorithm in the context of a study on NADP(H) metabolism in *E. coli* [49]. A manual evaluation of 280 relevant gene mentions in this study revealed that essentially all of the family assignments could be attributed to the EVEX gene family disambiguation method. GenNorm could only assign an Entrez Gene identifier to less than 1% of these gene mentions, owing to difficulties in resolving the organism and specific substrain in sufficient detail. Such precise resolution is not needed for gene family assignment, because genes across substrains are likely to fall into the same gene family. These results indicate that GenNorm works well in general, but may have specific problems to assign an organism when many strains/substrains are available. In such specific cases, the benefits of adopting a combined strategy for family assignment are apparent. In a notable contrast with the findings on the BioCreative III test set, the final manual evaluation of the combined family assignment algorithm in the *E. coli* study measured a precision of 84%, recall of 89%, and F-score of 87%.

In addition to illustrating the benefits of the specific combination strategy considered here, these results demonstrate how access to different layers of normalization granularity for the events extracted from text make it possible to create various combinations of the different normalization strategies according to the specific use-case, selecting either for high recall or high precision.

### Applications of normalized events

As demonstrated above, many of the algorithms applied in this study can be evaluated separately in terms of precision, recall and F-score. However, performance rates may vary drastically according to the domain or evaluation setup, as revealed by the normalization evaluation. Additionally, due to the complex interplay of the various components in the text mining pipeline and due to the influence of confidence-based filtering, the usability of this data as a whole can only be assessed within specific real-world use cases. In this section, a few promising example applications of this resource are illustrated, including data integration, database curation and pathway reconstruction.

#### Database integration

To demonstrate the application of the text mining data in the context of database curation, the data was integrated with experimental information from STRING v.9, a rich resource of protein associations incorporating data from many major domain databases, including high-throughput experiments, computationally inferred annotations, and manually curated pathways [1]. We first extracted from STRING all high-confidence protein pairs (using the score 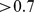 threshold suggested in STRING documentation) that identified at least one direct database source supporting the validity of the pair. With the goal of evaluating the possible integration of textual information with experimental data, we have excluded the resources that only contain computationally predicted relations, as well as STRING text mining co-occurrence data. We then mapped the STRING DB internal protein identifiers to Entrez Gene identifiers, obtaining 145K unique unordered protein pairs. These were then compared to the text mining predictions, reporting the results, broken down by source DB in STRING, in Table 6.

**Table 6:** Data integration analysis.

Database	Total # of pairs	Text mining match	Coverage
PID	998	820	82%
HPRD	1,057	694	66%
DIP	4,085	1,738	43%
GRID	28,735	8,346	29%
KEGG	72,620	19,739	27%
MINT	13,805	2,851	21%
IntAct	10,281	1,984	19%
Reactome	7,871	1,402	18%
BIND	6,453	1,135	18%
BioCyc	810	25	3%

Number of unique high-confidence protein pairs in STRING, and the proportion of these pairs for which an event is found through text mining.

A very broad variation in coverage is observed, ranging from over 80% for the PID database, to just a few percent for BioCyc. This broad variation is expected: the PID database, for example, consists solely of manually curated associations and requires literature support, while BioCyc additionally contains many sequence-based, computationally predicted associations, which are not expected to substantially overlap with existing literature. The high recall against fully curated databases like PID serves as an indirect verification of the text mining system and illustrates the potential of text mining for applications in database curation support. We further note that the recall numbers found here are expected to rise as more full-text articles become open access and thus available for text mining.

#### Pathway curation

**Figure 5:**
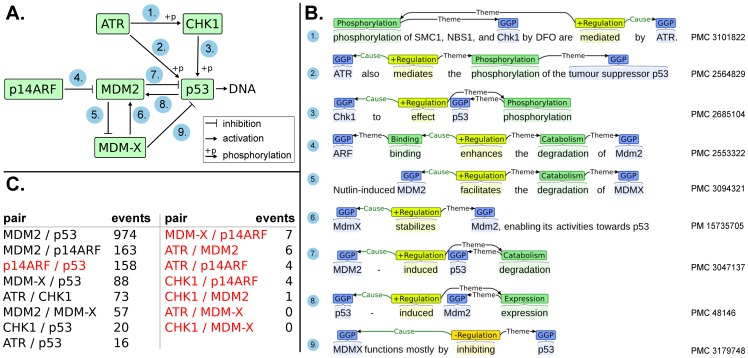
Events mapped to biomolecular pathways from KEGG. A) Close interactions of p53 from KEGG pathway hsa04115. B) The highest confidence predicted event from EVEX for each directed KEGG association. All are correct and correspond to the KEGG interaction type. Event visualizations were made with stav [27]. C) The number of events in the text mining dataset for each undirected protein pair. Pairs not corresponding to a direct molecular interaction in *A)* are shown in red.

Another promising application of event extraction involves assisting pathway curation and analysis. While previous work on this topic has involved small-scale evaluation with manual mapping between gene symbols and identifiers [50], our novel addition of gene normalization data now allows direct evaluation of the textual events in the context of pathway curation at the scale of the entire available literature. To illustrate this use case, we have analysed a subsection from a well-known KEGG pathway [21], evaluating whether it can be recreated using text mining information. The target pathway is the *human p53 signaling pathway*, illustrated in Figure 5 A. This subsection was selected at random from the full pathway prior to the analysis.

For each pair of proteins in the KEGG *p53* interaction subnetwork, we have taken all text mining events linking those two proteins. Note that since these gene IDs refer to human genes, this successfully restricts the textual result set to human biology. The number of events for each protein pair is shown in Figure 5 C. While most events correspond to direct physical interactions, statements in literature can also refer to indirect regulatory control, and this could be a source for many of the events on e.g. *p14ARF* associations with *p53*.

For each KEGG interaction that connects a directed pair of proteins, we have further selected the event with the highest confidence score. These events are shown in Figure 5 B. Notably, all of the highest confidence events are correctly extracted and equivalent to the KEGG interaction type. Further, the PMC articles are prevalent as a source for the highest confidence events, once more underlining the importance of mining full-text articles. Additional details on this evaluation as well as the relevant data on the full *p53* pathway are depicted in data S1.

This example directly demonstrates the benefits of our data for pathway reconstruction and providing textual evidence for known interactions. However, this approach could also straightforwardly be applied to assist pathway curators in the search for candidate interactions established in the literature but not previously incorporated in pathway models.

## Conclusions

We have presented a text mining analysis that combines structured event extraction with gene normalization – two major lines of research in the BioNLP community – to process all PubMed abstracts and all open access full texts in PubMed Central. The applied text mining pipeline represents the state of the art, confirmed by the results of community-wide shared task evaluations. The resulting dataset contains 40 million biomolecular events among 76 million gene and protein mentions from over 5000 species. Covering protein metabolism, fundamental molecular events, regulatory control, epigenetics, post-translational modifications and protein domains and complexes, this resource is unprecedented in semantic scope, literature coverage, and data retrieval facilities.

In this study, we have extended the gene normalization step of the text mining pipeline to produce mention-level results rather than only document-level ones. Additionally, we integrated a canonicalization algorithm and gene family definitions from HomoloGene, Ensembl and Ensembl Genomes, enabling a multi-level normalization strategy. We have demonstrated that such an integrative normalization method is useful to resolve cases where gene families are mentioned rather than individual genes, and those where the exact organism or substrain is difficult to distinguish. Further, specific normalization combinations allow selecting for either high recall or high precision of the results. By publicly releasing all our data, we hope to encourage the exploitation of this information also in other text mining studies and frameworks.

The detailed evaluations presented in this study illustrate that there is still room for improving the algorithms behind the various text mining components. However, by integrating these components into a unified pipeline and running them on the whole of PubMed and PubMed Central open access documents, instance-level recall issues are minimized and the chances of finding any specific piece of biologically relevant information are significantly increased. Indeed, it is sufficient to only extract a certain biological event once for it to be useful in a specific case-study. Additionally, we have shown that the confidence values, automatically assigned to the textual event predictions, can be used for selecting high-precision subsets of the data at various thresholds. Together, these opportunities were illustrated on example use-cases, obtaining high recall against manually curated databases such as PID. Further, we have shown on a specific pathway example that text mining data allows for accurate extraction of relevant literature and interaction partners of uniquely identified genes. These promising results demonstrate the potential of text mining applications in database curation and knowledge summarization.

All data produced in this study has been integrated into the EVEX resource (http://evexdb.org), and will be regularly updated to include the latest findings from literature. Further, this data is freely available through bulk downloads (http://evexdb.org/download/) as well as through a new programmatic interface (http://www.evexdb.org/api/v001/), providing a unique large-scale dataset for use in various bioinformatics studies. The data is further distributed with the original sentences it was derived from, allowing its use in other text mining studies.

In future work, we plan on targeting additional biological use-cases to further evaluate and improve on our integrative methods. In the framework of these future studies, we plan on improving the EVEX website and API to accommodate also researchers outside the field of BioNLP. Finally, we will build upon the normalization evaluations presented in this study to further enhance gene normalization in the context of event extraction, performing additional evaluations on a recently released mention-level corpus, as well as augmenting the textual events with information derived from figures and tables.

## Supporting Information

Data S1 This file provides additional details on the pathway curation use-case, which describes a subsection of the *human p53 signaling pathway*. In this supplemental file, the data on the full *p53* pathway are also provided.
(XLS)
